# The Current of Consciousness: Neural Correlates and Clinical Aspects

**DOI:** 10.1007/s11910-023-01276-0

**Published:** 2023-06-12

**Authors:** Garrett Friedman, Katherine W. Turk, Andrew E. Budson

**Affiliations:** 1 Center for Translational Cognitive Neuroscience, VA Boston Healthcare System, 150 S. Huntington Ave., Jamaica Plain, Boston, MA 02130 USA; 2 Department of Neurology, Boston University Chobanian & Avedisian School of Medicine, Boston, MA USA

**Keywords:** Consciousness, EEG, Event-related potentials, P3B, VAN, Memory theory

## Abstract

**Purpose of Review:**

In this review, we summarize the current understanding of consciousness including its neuroanatomic basis. We discuss major theories of consciousness, physical exam-based and electroencephalographic metrics used to stratify levels of consciousness, and tools used to shed light on the neural correlates of the conscious experience. Lastly, we review an expanded category of ‘disorders of consciousness,’ which includes disorders that impact either the level or experience of consciousness.

**Recent Findings:**

Recent studies have revealed many of the requisite EEG, ERP, and fMRI signals to predict aspects of the conscious experience. Neurological disorders that disrupt the reticular activating system can affect the level of consciousness, whereas cortical disorders from seizures and migraines to strokes and dementia may disrupt phenomenal consciousness. The recently introduced memory theory of consciousness provides a new explanation of phenomenal consciousness that may explain better than prior theories both experimental studies and the neurologist’s clinical experience.

**Summary:**

Although the complete neurobiological basis of consciousness remains a mystery, recent advances have improved our understanding of the physiology underlying level of consciousness and phenomenal consciousness.

## Introduction

### Definition

The term “consciousness” often carries different definitions. In this article, we define “consciousness” as having two core components:

1. *Phenomenal consciousness*: our personal, subjective experience of the present moment [1]. Understanding the neural basis of phenomenal consciousness is referred to by Chalmers as “The hard problem” [2].
2. *Level of consciousness*: the state of wakefulness assessed by various scales described in terms such as comatose, stuporous, obtunded, delirious, confused, and alert. Level of consciousness can be thought of as a volume dial, upon which phenomenal consciousness is dependent.

### Theories of Consciousness

There are at least twenty-two supported neurobiological explanations [see REF 3•] for the basis of consciousness. In this review, we will reference a few of the major theories.

*Higher Order Theory* (HOT) claims that consciousness depends on meta-representations, representations that occur higher in a processing hierarchy. For example, whereas the lower order representation of visual stimulus of a painting is represented in visual cortex as “red and green areas,” it only achieves consciousness when relayed to higher areas—specifically the pre-frontal cortex—allowing for the self-conscious statement, “I am having the experience of seeing a painting” [4, 5].

*Global Workspace Theory* (GWT) postulates a centralized resource shared by multiple independent threads of processing. Mental states are conscious if and only if they are available to a wide range of cognitive processing, which depends on a frontoparietal network accessible to multiple local processing networks throughout the cortex [6–8].

*Integrated Information Theory* (IIT) suggests that consciousness is defined by a system’s structural capability to produce irreducible, integrated information, a measurable and unidimensional quantity referred to as “Φ.” The brain regions with the highest Φ value are thought to be a temporo-parieto-occipital area, the “posterior hot zone” [9, 10].

*Local Recurrency Theory* (LRT) argues that recurrent processing in sensory cortices is sufficient to generate conscious experience while parietal and frontal regions are required only for reports and judgements pertaining to a stimulus [11, 12].

The *Memory Theory of Consciousness* (MToC) proposes that the phenomenonal consciousness evolved from, and functions as a part of, episodic memory and other explicit memory systems (sensory, working, and semantic memory) [13••]. This theory can explain long-standing puzzles in consciousness such as postdictive effects—the finding that conscious perception of a stimulus can be affected not only by conditions *preceding* a stimulus, but also by the conditions *following* a stimulus. The entire cerebral cortex and hippocampus function together to serially integrate and time stamp parallel unconscious brain processes to form a linear, coherent stream of conscious experiences. This theory proposes that the experience of consciousness is actually a memory, preceded temporally by unconscious processes of sensation, decisions, and/or actions. The theory also proposes that different cortical areas produce different aspects of consciousness, each with its own neural correlate [13••].

Each of these theories works towards understanding the neural correlates of consciousness—the neurobiological mechanisms necessary and sufficient to generate a conscious experience. A better understanding of consciousness and its neurobiology may improve the clinical care of patients with disorders of consciousness, including not only anoxia and severe traumatic brain injury but also delirium, epilepsy, migraine aura, cortical strokes, and cortical dementias.

## Clinical Metrics of Levels of Consciousness

Level of consciousness, that is, the state of wakefulness, is commonly defined clinically through descriptors of psychomotor activity in response to stimulus. The Glasgow coma scale (GCS) is perhaps the most widely used clinical scale of consciousness, favored for its three metric simplicity (eye opening, verbal response, motor response). However, in recent years, it has drawn criticism for lack of content validity, standardization, and inter-examiner reliability [14, 15]. A simpler measure favored in the intensive care unit (ICU) setting is the Richmond Agitation-Sedation Scale, which plots wakefulness on a single dimension from combative behavior to complete unresponsiveness [16]. Another scale with high inter-examiner reliability, content validity, standardization, and prognostic capability is the Coma Recovery Scale Revised (CRS-S) [17–19]. See [REF 6] for other scales.

Patients may, however, be clinically unresponsive and maintain electroencephalographic (EEG) or functional MRI (fMRI) activity that suggests underlying consciousness [20–23]. In a large, ICU-based study of brain activity, Claasen et al. used EEG patterns to detect occult brain activity in response to spoken motor commands in patients with acute brain injury [24••], observing that about 15% of clinically unresponsive patients have a “conscious” EEG pattern in response to spoken commands. Follow-up investigations found that 41% of patients with covert consciousness make a complete recovery versus only 10% of patients without covert consciousness [25•].

## EEG Activity as a Correlate of Consciousness

EEG is the gold standard in determining the sleep-wake phase and is a useful tool in assessing the level of consciousness. The Bispectral Index (BIS) is used by anesthesiologists to assist in the titration of anesthetics. Raw EEG data is collected from four electrodes on the forehead and analyzed by a proprietary, algorithm which outputs a unidimensional score, the BIS, which quantifies the level of wakefulness from 0 (comatose—flat line EEG) to 100 (awake—low amplitude, fast frequency) [26].

EEG frequency ranges and measures of connectivity have been considered as potential correlates of phenomenal consciousness (or at least aspects of it). The alpha frequency (8–12 hz), canonically considered to be an idling rhythm, has recently been thought to have a functional role in the inhibition of task-irrelevant information [27–30]. Pre-stimulus alpha activity predicts poor conscious perception; occipital alpha power might filter out which stimuli will be perceived consciously, and which stimuli will be deemed “task irrelevant” and ignored [31–34].

For example, Krasich et al. showed that about halfway between the maximum positive and negative peaks of occipital alpha activity, a stimulus is mostly likely to be consciously perceived [35••]. In the post-stimulus period, a drop in parietooccipital alpha power has been suggested as a marker of conscious perception [36]. Hutchinson et al. studied a paradigm of inattentional blindness and confirmed that alpha power 250–500 ms post stimulus is negatively associated with conscious perception [37].

## ERP Activity as a Correlate of Consciousness

Event-related potentials (ERPs) are EEG changes time locked to sensory, motor, or cognitive events that provide a means to study electrophysiologic correlates of mental processes. ERPs provide excellent temporal resolution and are a practical (non-invasive, relatively inexpensive) method of studying cognitive functions [38].

The conscious perception of a stimulus depends on a variety of factors including internal features of the stimulus itself (such as its salience). External factors that affect stimulus perception include observer state (such as attentional) and context (such as masking) which may affect perception preceding, simultaneously, or following the stimulus [39]. Each of these factors can be experimentally controlled and modified in ERP study paradigms to determine the brain signals that are together necessary and sufficient for conscious perception—the neural correlates of consciousness [40].

Early ERP components, peaking within 100 ms after stimulus, are known as “sensory” or “exogenous,” as they depend mostly on the physical parameters of the stimulus itself. The middle and late components, generated 100–300 ms and 300–600 ms after stimulus, respectively, reflect stimulus evaluation and are termed “cognitive” or “endogenous” potentials [41].

### Early ERP Components (P50, N100, and Others)

In their historic work, Libet et al. reported in 1967 that early ERP sensory evoked potentials over the primary somatosensory cortex are not always sufficient to cause perceptual experience [42]. This finding, replicated and expanded upon by others [43–46], started the search for other early potentials to determine the first signals sufficient to predict sensory experience, also known as perception.

### Middle ERP components (P100, N140, ERN/Pe)

#### P100

The P100, a positive occipital ERP component occurring 100 ms after stimulus presentation, has been implicated as the earliest ERP involved in conscious perception and associated tasks [39, 47, 48]. Other work, however, found the P100 in both perceived and unperceived conditions [49].

#### N140

The N140 is a negative ERP peaking around 140 ms. Multiple studies highlight the N140 as important for perception but there is debate as to whether the signal should be interpreted as awareness, perception, detection, or attention [50–52]. Given the prolongation of the N140 in patients with clinical deficits in contralateral spatial attention, we favor this specifically as a marker of somatosensory hemi-attention rather than conscious perception [53].

### Late ERP Components (late Pe, P300, VAN)

Our review of early and middle ERP components suggests that they are mainly correlated with attention or unconscious sensory processes. Later, ERP components may be more likely to represent true neural correlates of consciousness.

#### Positivity Performance Error (Pe)

The late positivity performance error (Pe) component (400–600 ms) has been associated with conscious awareness of error detection [54].

#### P300

The P300 is a posterior ERP observed 290–450 ms after a stimulus [55]. Historically, the P300, which includes components P3a (central maximum) and P3b (parietal maximum), has been thought to represent a gold-standard marker of conscious perception [56–58]. Recently, however, the P300 has been favored to specifically represent post perceptual processing, rather than phenomenal conscious awareness [See REF 59, 60• for in-depth review].

#### VAN

The visual awareness negativity (VAN) is a negative difference wave, observed 200 ms after stimulus presentation, most prominently over occipitotemporal sites, which differentiates aware from unaware states [61]. The VAN may be the earliest reliable electrophysiologic marker that correlates with aware perception after the presentation of a visual stimulus. There is an analogous auditory awareness negativity leading to recent proposals to consider these potentials as parts of a broader termed “perceptual awareness negativity” [62].

## Connectivity as a Correlate of Consciousness

In the conscious patient, a TMS pulse sets off a complex chain of events as information reverberates through cortical brain regions, but in the unconscious patient, a similar pulse may induce only a short-lived local effect [63–67]. This finding supports and provides a testable metric for IIT that applies a connectivity approach to evaluate levels of consciousness.

Studying EEG recordings in patients with diminished levels of consciousness as determined by GCS, Toker et al. proposed that state of consciousness correlates with the proximity of slow cortical oscillatory potentials to a critical pattern described as the edge-of-chaos [68]. In a serial awakening paradigm, exploring EEG phase connectivity as a correlate of consciousness, patient states were differentiated across sleep stages by measures of EEG phase connectivity in posterior brain regions [69•].

Similarly, using fMRI, Demetrezi et al. found that compared to healthy controls, those with decreased level of consciousness showed impaired baseline connectivity in bilateral executive control, default mode, and auditory networks [70]. Luppi et al. employed resting-state fMRI with measures of integration and entropy to identify consciousness-specific patterns of brain function including temporal states of high integration and functional diversity [71••].

## Aspects of Consciousness

If we assume that consciousness is unitary, prompting us to look for a single neural correlate of consciousness, then it is difficult to make sense of these divergent results from various studies using different measures of brain physiology. If, however, we take the approach suggested by the MToC—that there are various aspects of consciousness such as visual, auditory, emotional, phonological, motor, and more—then each study may reveal the neural correlate of consciousness for one aspect of consciousness but not others [13••]. This modular view of consciousness is entirely consistent with the disorders of consciousness encountered daily by the neurologist.

## Disorders of Consciousness

Classically, this group included only disorders that directly impact the level of consciousness, such as TBI and cardiac arrest. Following the MToC, however, we broaden this definition to include disorders that affect phenomenal consciousness [13••].

### Seizure

Epileptic seizures offer a window into the requisite anatomy and function for maintaining consciousness as different seizure subtypes affect consciousness differently. A generalized onset seizure includes bilateral motor involvement, loss of consciousness, and involvement of bilaterally distributed networks [72]. The fact that complete loss of consciousness in seizure requires generalized involvement of both hemispheres argues strongly that level of consciousness is a diffuse cortical phenomenon, consistent with MToC but not HOT, GWT, IIT, or LRT. Focal seizures are subclassified as impaired awareness or unimpaired awareness [73]. Focal seizures with altered awareness also impair phenomenal consciousness, as manifested by, for example, amnesia, automatisms, and behavioral arrest [74].

### Migraines

Although migraine does not generally affect the level of consciousness, Budson et al. argued that migraine aura is, in fact, a focal disruption of phenomenal consciousness [13••]. Rarely, in the case of the basilar sub-type, migraine can affect the level of consciousness, causing confusion. More commonly, migraine aura results in the impairment of various aspects of phenomenal consciousness, such as visual associated with posterior onset of cortical spreading depression [75]. Positive followed by negative visual phenomena (scintillating scotoma) are common changes associated with focally impaired conscious awareness.

### Strokes and Other Discrete Brain Lesions

Stroke, discrete in border and stable over time, is the prototype brain lesion and offers a means to evaluate the neuroanatomic basis of consciousness. We will first discuss how strokes affect the level of consciousness, and then how strokes can affect various aspects of phenomenal consciousness.

At onset, level of consciousness is impaired in about 36% of patients with large hemispheric infarcts and 14% of stroke patients as a whole [76, 77]. Clinical attention to level of consciousness is critical as early decreased consciousness may portend a 2.2-fold risk of in-hospital mortality [37]. Outside of infarcts that affect wide distributions of cortex (directly or indirectly through edema and hydrocephalus), the best described lesions affecting the level of consciousness are those within the reticular activating system (bi-thalamic and brainstem) [78–80]. In a highly powered brain mapping study of penetrating head lesions, no individual cortical regions were associated with loss of consciousness while lesioned region connectivity to the dorsal midbrain (i.e., reticular activating system) did correlate with loss of consciousness [81•].

Cortical strokes, especially when occurring bilaterally, may cause impairments to various aspects of phenomenal consciousness. For example, unilateral occipital infarcts impair hemifield visual consciousness and bilateral occipital infarcts may cause cortical blindness and a complete loss of conscious visual perception (despite some intact unconscious function) [82]. However, no single or bilateral cortical infarct, unless involving most of the cortex, leads to permanent unconsciousness. If there were a “hot zone” of consciousness (as suggested by HOT, GWT, IIT, and LRT), there should be a cortical stroke syndrome that devastates consciousness; no such cortical lesion has been described. By contrast, MToC suggests that cortical strokes will impair aspects of phenomenal consciousness without affecting the level of consciousness.

### Delirium

Delirium is a syndrome characterized by an alteration in attention, awareness, and cognition [83•]. Budson et al. suggested that delirium is a state of individuals being awake but unconscious, noting that they do not appear to have conscious control of their own actions [13••]. The prevalence of delirium in hospitalized patients is high: up to 23% of hospitalized patients and 88% of palliative care inpatients [84, 85]. Despite the high prevalence of delirium, the underlying pathophysiology of delirium is unclear. Recent studies have approached the neural substrate of delirium from a connectivity and functional state perspective. Fleishmann et al. suggested that theta band hyperconnectivity and alpha band dysconnectivity may underly delirium [86]. Montfort et al. support the theory of disintegrate resting state in delirium and revealed a specific loss of function in the posterior cingulate cortex among delirious patients [87].

### Dementia

Although not generally considered a disorder of level of consciousness, dementia almost always affects aspects of the phenomenal conscious experience [13••]. Recent studies have attempted to answer the question of how conscious experience might be affected by the dementias.

Patients with Alzheimer’s disease demonstrated poor ratings in visual imagery, auditory imagery, and spatiotemporal specificity—all core components of the phenomenal conscious experience [88]. In a study of ERPs, latency of the P300 (a candidate correlate of consciousness discussed above and supported by MToC as a marker of one aspect of phenomenal consciousness) showed the highest correlation with Alzheimer’s disease pathology, supporting previous studies using the P300 as a marker of cognitive decline [89–91].

Corticobasal syndrome and alien-limb phenomenon present a disconnection between action and volitional, conscious control. In 2019, the first MRI-based group analysis of alien limb phenomenon in corticobasal degeneration revealed that CBS with alien-limb phenomenon can be differentiated from CBS patients without alien-limb syndrome based on structural differences in the cingulate gyrus as well as the frontomedian cortex, post central gyrus, and temporoparietooccipital regions [92•]. This finding supports previous literature that the anterior cingulate gyrus is critical for conscious control of motor functions [93].

Not all effects of dementia dampen consciousness. In some patients with FTD, a domain of consciousness can be augmented. The fascinating emergence of artistic creativity in some FTD patients has been described since 1998 [94]. Recent systematic review suggests that the relative preservation of the occipital cortex, which may be less inhibited by degenerating frontotemporal systems, is strongly correlated with visual artistic creativity [95].

Lastly, the absence of level of conscious deficits in neurodegenerative diseases raises questions about the validity of some theories of consciousness. If meta-cognition in the frontal lobe is essential for consciousness, as stated in HOT, one would expect that patients with moderate FTD to become unconscious and lack any conscious awareness of their surroundings. Similarly, if the posterior brain and its connectivity is the center of consciousness as maintained by proponents of IIT and RPT, then patients with posterior cortical atrophy would be expected to have impairments beyond the visuospatial domain and become unconscious. Impairments in patients with dementia, therefore, are best explained by MToC, which argues for impaired aspects of phenomenal consciousness with dementia.

## Conclusions

Although the complete neurobiological basis of consciousness remains a mystery, recent advances have improved our understanding of the disorders of levels of consciousness and of aspects of phenomenal consciousness. The idea espoused by Budson et al. in the MToC—that the substrate of consciousness is the cerebral cortex, which enables various aspects of phenomenal consciousness—is consistent with both the neurological literature and the experience of clinical neurologists [13••]. Different ERPs likely correspond to different aspects of phenomenal consciousness—not all of consciousness—which may explain some of the disagreements in the literature. Neurological disorders that disrupt the reticular activating system can affect the level of consciousness, whereas cortical disorders from seizures and migraines to strokes and dementia may disrupt phenomenal consciousness. We believe that consideration of these neurological disorders argues against local theories of consciousness and best support theories of consciousness as distributed through a large cortical network, such as MToC. The future of consciousness research will be furthered by the application of experiential paradigms assessed by EEG, ERP, and fMRI to patients with disorders of consciousness. Such research may improve clinical care and reveal the neurological basis of consciousness.
